# 974. Objective Evaluation of Short-term Effects of Paraffin Wax on Hypertrophic Scars

**DOI:** 10.1093/jbcr/irag033.571

**Published:** 2026-04-06

**Authors:** Zoë Edger-Lacoursière, Yumi Amal Bellali, Stéphanie Jetté, Mathieu Delisle, Aude Pinard-LaRoche, Bernadette Nedelec

**Affiliations:** Villa Medica Rehabilitation Hospital and McGill University, Montréal, QC; McGill University, Montréal, QC; McGill University, Montréal, QC; McGill University, Montréal, QC; McGill University, Montréal, QC; McGill University, Montréal, QC

## Abstract

**Introduction:**

Paraffin wax is applied to scar tissue for its proposed benefits of increasing skin.

pliability and relieving pain. However, limited studies have objectively assessed hypertrophic scar (HSc) response. This study examined immediate HSc changes after paraffin treatment compared with intra-individual controls.

**Methods:**

A quasi-experimental, within-subject, evaluator-blinded, pre-post design was used. Two homogeneous HSc were identified per participant: one treated with paraffin wax and the other with mineral oil for 20 minutes. Normal skin also received paraffin wax treatment to assess scar-specific effects. Objective measures included elasticity (Cutometer), erythema and melanin (Mexameter), transepidermal water loss (TEWL) (Tewameter), thickness (High-frequency Ultrasound), and self-reported pain, itch, stiffness, and overall scar impression.

**Results:**

Thirty-nine burn survivors were recruited; 35 completed the study, ensuring an adequate sample size. Paired t-tests showed increased elasticity and erythema in paraffin-treated HSc (*p*=.04; *p*<.01 respectively) and in normal skin (*p*=.03; *p*<.01 respectively), decreased pigmentation in paraffin-treated normal skin (*p*=.02), and decreased TEWL in the mineral oil-treated control scars (*p*=.01). ANCOVA of post-treatment values, controlling for baseline, revealed significant increases in erythema and TEWL in paraffin-treated versus control scars (*p*=.03; *p*<.01 respectively). There was no significant difference in skin elasticity between groups (*p*=.65). At baseline, most participants reported no scar pain (80% in both groups) and no itch (60% in the paraffin group vs. 68.6% in the control group). Among participants who reported pain or itch, they reported greater improvement in paraffin-treated HSc than controls, though controls also improved across all items.

**Conclusions:**

Although paraffin wax significantly increased elasticity in both HSc and normal skin, its effect did not significantly differ from mineral oil treated scar, suggesting that improvements in elasticity may be attributable to mineral oil. Nevertheless, paraffin demonstrated a greater improvement on all self-reported outcomes. Future studies should target participants with elevated baseline scar pain and itch to ensure adequate power for detecting these effects and examine the benefits of mineral oil across time.

**Applicability of Research to Practice:**

Findings suggest that improving burn survivors’ self-reported outcomes may be more important indicators for paraffin wax treatment than increasing scar elasticity. Clinicians can use these findings to guide treatment selection to optimize rehabilitation outcomes.

**Funding for the study:**

N/A.

